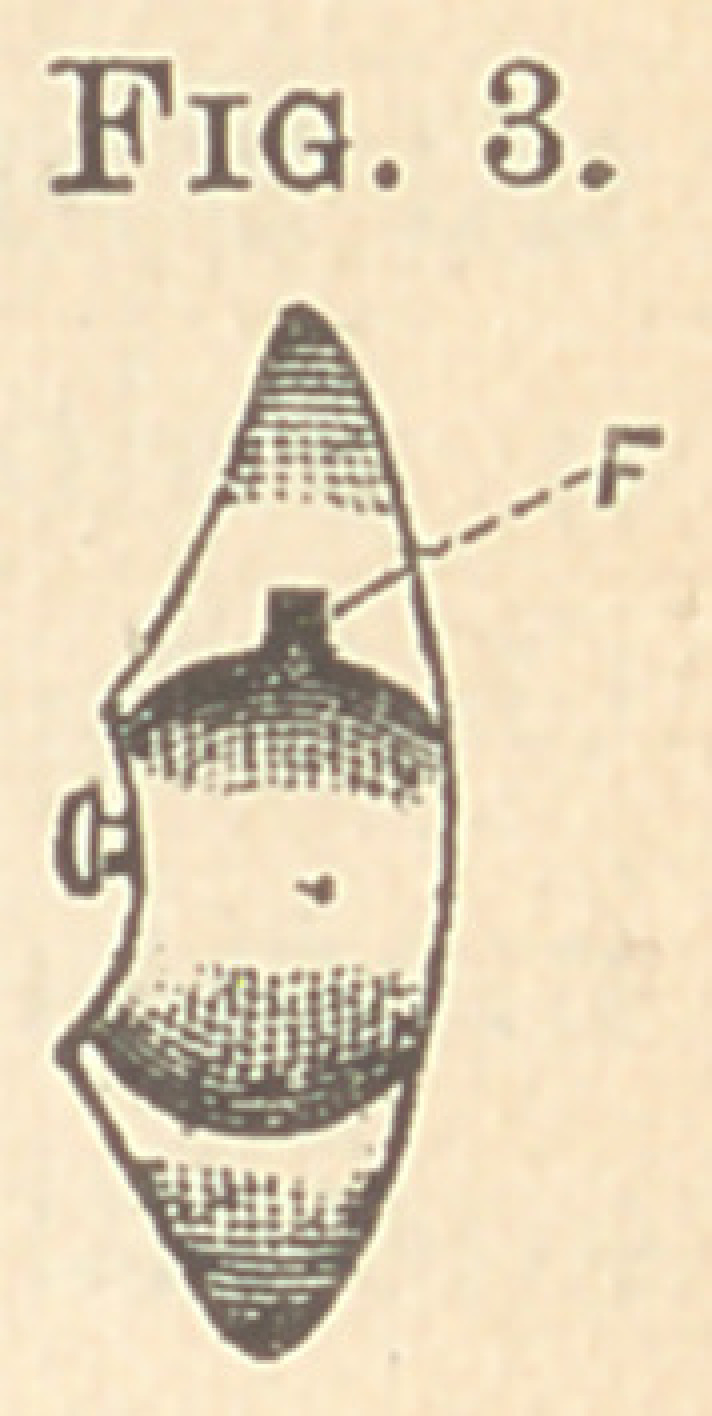# Domestic Correspondence

**Published:** 1889-08

**Authors:** H. A. Keely

**Affiliations:** Portland, Maine


### Domestic Correspondence.

To the Editor:

Every one who has occasion to place a filling in a porcelain tooth has experienced the dissatisfactory working of the diamond drill. The objections are, first, from the length of time consumed, as a diamond drill cannot be hurried; second, from the expense, owing to the frequent breaking of the drill; and third, the unsatisfactory edge produced.

The method I would advocate is as follows: To form a cavity in a central on the mesio-labial surface, first take an ordinary corundum wheel with round edge and of sufficient thickness to give the required length of cavity,—that is, from as near the cervix to the cutting edge as is desired.

Fig. 1.

Now cut from the mesial surface directly into the tooth until you have the cavity deep enough to show the filling when in place, the desired amount on the labial surface. The labial aspect of the tooth will present the appearance seen in Fig. 1, with a well-formed edge; the lingual aspect the form shown in Fig. 2, giving a distinct shoulder at *a* and *b*. Now, with a copper disk, fairly thin, cut in at *a* and *b* in the directions of *c* and *d* respectively. In this way the filling may be held from falling out either upward, downward, or towards the labial surface.

Fig. 2.

To prevent its moving backward, make undercuts, still using the copper disk, just anterior to *a* and *b* respectively. The undercut made anterior to *a* will be entirely on the mesial surface, and is represented at *f*, Fig. 3. It will be covered by the rubber when in place. That at *e* may be cut well in, finishing off the filling in the concavity on the lingual side just below the shoulder, which is beneath the pins. This method of retaining the filling is essentially a system of dovetails. It is advisable to bend the pin, approximating the cavity, back out of danger of injury by the grinding.

Fig. 3.

After forming the cavity, invest in plaster, for convenience in handling, and fill. Then take out of plaster and finish. A filling put in by this method will be firm in place and present a perfect edge, which latter it is quite impossible to obtain by means of the diamond drill. Of course the edge, posteriorly, will be irregular in outline, but that is covered by the rubber when the tooth is vulcanized in place.

H. A. Keely, D.M.D.

Portland, Maine.

## Figures and Tables

**Fig. 1. f1:**
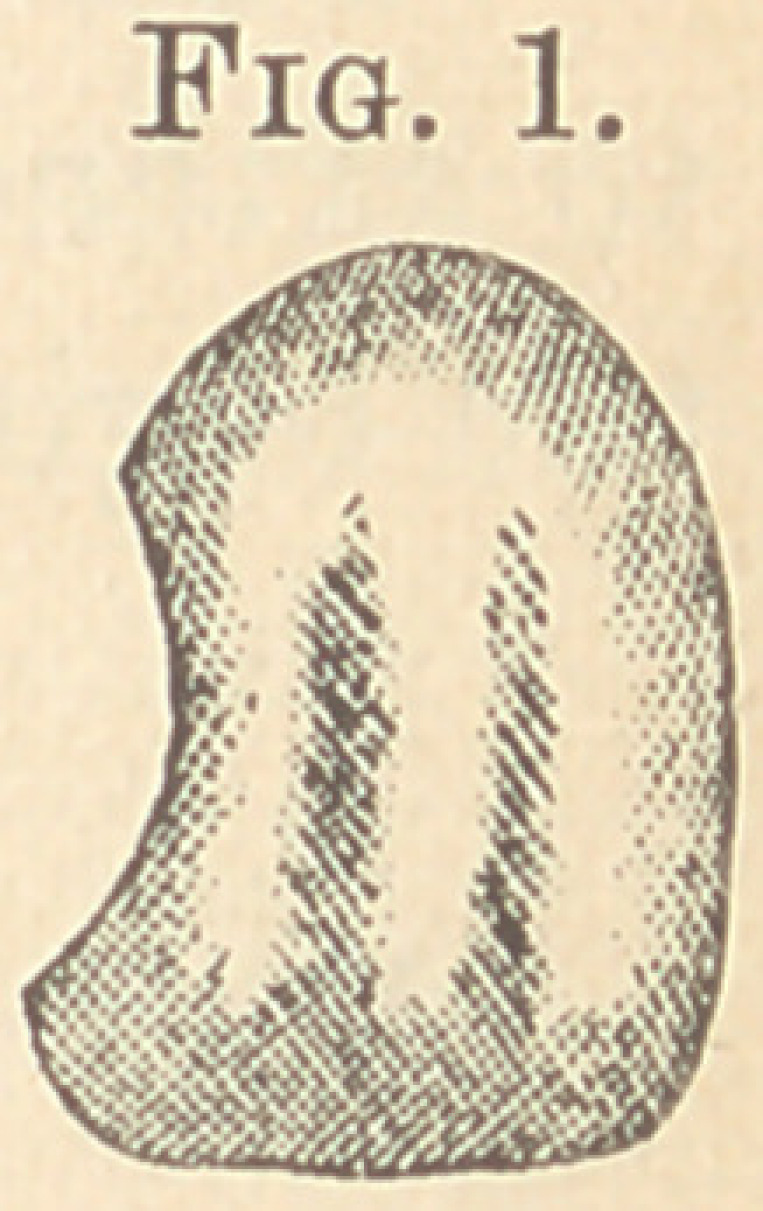


**Fig. 2. f2:**
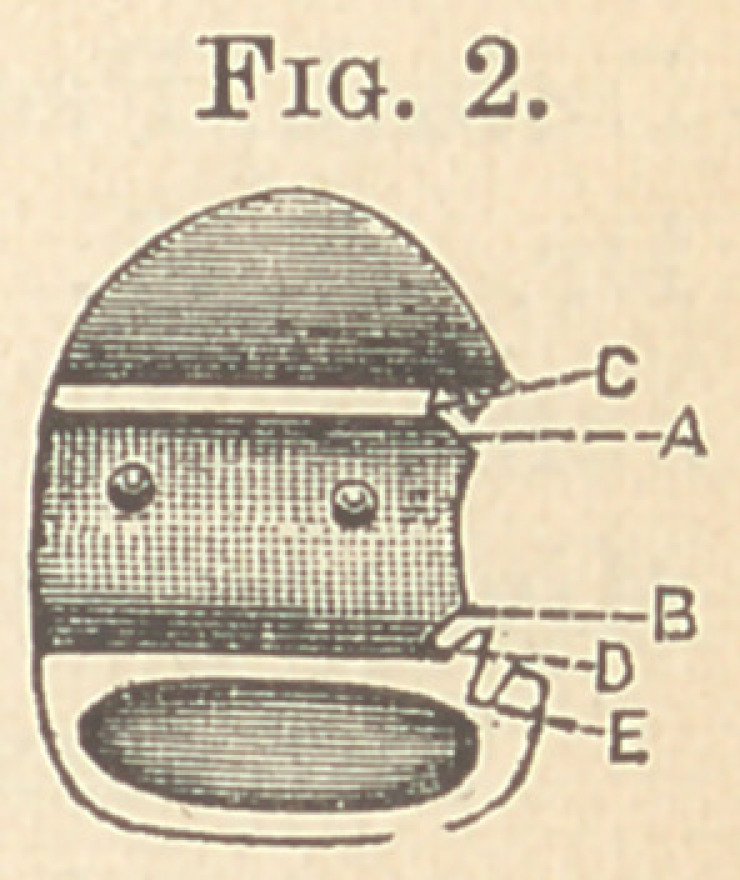


**Fig. 3. f3:**